# Biomimetic materials

**DOI:** 10.3762/bjnano.2.16

**Published:** 2011-03-10

**Authors:** Wilhelm Barthlott, Kerstin Koch

**Affiliations:** 1 Nees Institute for Biodiversity of Plants, Rheinische Friedrich-Wilhelms University of Bonn, Meckenheimer Allee 170, 53115 Bonn, Germany; 2 Rhine-Waal-University of Applied Sciences, Landwehr 4, 47533 Kleve, Germany

Life is a specific characteristic of our planet. The diversity of life, biodiversity, is one of the most fascinating phenomena. We know some 1.8 million different species, but all extrapolations show that probably 20 million or more species exist: We know less than 10% of the plants, animals and micro-organisms living on the planet Earth. The editors, who are both biologists, are well acquainted with these figures whilst for non-biologists this fact is most surprising and even unbelievable. The process of life started more than one billion years ago, it is a continuous process of evolution, of mutation and selection, of structures and materials. Put another way, this means one billion years of trial, error and optimization. The diversity of life thus created a breath taking diversity of materials and structures based on an extremely restricted number of elements.

It took a surprisingly long time until we understood what we can learn from one billion years of biological development work. No doubt, Daedalus and Icarus in Greek mythology copied biological models – without, however, great success. This is an unbroken tradition from past epochs to the era of Leonardo da Vinci up to the present day. However, systematic research of biological systems for a technical application began astonishingly quite late: Bionics and biomimetics became important only after 1960, and it was only in the new millennium they became worldwide disciplines with high potentials for innovation. An apparently simple observation can lead to new materials, structures and design principles, but the technical transformation and realization may take a much longer time. Our own example of the self cleansing abilities of lotus leaves clearly demonstrates this point: Published for the first time in 1976, patented as a technical feasibility in 1994, facade paints only appeared on the market in 1999.

Bionics concentrated in the first decades, in particular, on highly complex biomechanic problems: the hovering of humming birds and robot locomotion. Today a focal point is the incredibly specific diversity of biological materials, materials with micro and nano structures, often self-assembling. Another change of paradigm was caused by lotus leaves: surfaces and boundary layers. All interactions between an organism and its environment take place via its surface – be it the interaction between a solid (biological species), a liquid (e.g., water), a gas (e.g., air) and radiation (e.g., sunlight). Boundary layers and, in particular, superhydrophobic surfaces and their interactions with the environment were thus the focus of this Thematic Series on *Biomimetic materials*.

The most interesting phenomena happen on boundary layers: from the biosphere at the boundary layer of our planet down to the surfaces of lotus leaves or Salvinia water ferns. And these are only two out of the 20 million species which all have secrets to be revealed: Biomimetic materials provide innovative solutions for the design of a new generation of bio inspired functional materials.

Wilhelm Barthlott and Kerstin Koch

Bonn, Kleve, March 2011

